# Strabismus Promotes Recruitment and Degradation of Farnesylated Prickle in *Drosophila melanogaster* Planar Polarity Specification

**DOI:** 10.1371/journal.pgen.1003654

**Published:** 2013-07-18

**Authors:** Helen Strutt, Vickie Thomas-MacArthur, David Strutt

**Affiliations:** MRC Centre for Developmental and Biomedical Genetics and Department of Biomedical Science, University of Sheffield, Western Bank, Sheffield, United Kingdom; Harvard Medical School, Howard Hughes Medical Institute, United States of America

## Abstract

The core planar polarity proteins are required to specify the orientation of structures that are polarised in the plane of the epithelium. In the *Drosophila melanogaster* wing, the core proteins localise asymmetrically at either proximal or distal cell edges. Asymmetric localisation is thought to be biased by long-range cues, causing asymmetric complexes to become aligned with the tissue axes. Core proteins are then thought to participate in feedback interactions that are necessary to amplify asymmetry, and in order for such feedback interactions to operate correctly, the levels of the core proteins at junctions must be tightly regulated. We have investigated regulation of the core protein Prickle (Pk) in the pupal wing. The core protein Strabismus (Stbm) is required to recruit Pk into asymmetric complexes at proximal cell ends, and we report here that it also promotes proteasomal degradation of excess Pk, probably via a Cullin-1 dependent process. We also show for the first time that Pk is farnesylated *in vivo*, and this is essential for Pk function in the wing. Notably, farnesylation of Pk is necessary for it to be recruited into asymmetric complexes and function in feedback amplification, probably by reinforcing weak direct interactions between Stbm and Pk. Furthermore, farnesylation is also required for Stbm to promote proteasomal degradation of Pk. We propose that Stbm recruits farnesylated Pk into asymmetric complexes, but also promotes degradation of excess Pk that would otherwise perturb feedback amplification.

## Author Summary

The core planar polarity proteins are responsible for polarising structures in the plane of epithelia. For example in the fly wing, the core proteins are required for cells to make hairs that point towards the distal end of the wing. The core proteins localise asymmetrically in wing cells, either at the distal cell end where the hair emerges or at the opposite cell edge. To establish this asymmetric localisation the core proteins must undergo feedback interactions with each other, and it is thought that for feedback to operate correctly, the amounts of the core proteins at junctions must be limiting. We show that the core protein Prickle is modified by a farnesyl lipid molecule. This modification is essential for it to associate with cell membranes where it can interact with another core protein, Strabismus. Interaction with Strabismus allows Prickle to participate in asymmetric complexes and feedback interactions, but Strabismus also causes degradation of excess Prickle. If Prickle doesn't interact with Strabismus, or if there is too much Prickle at cell membranes, asymmetric localisation of the other core proteins is compromised.

## Introduction

The Prickle (Pk) protein is one of the “core” planar polarity proteins which are necessary to polarise cells in the plane of epithelia in *Drosophila melanogaster* and vertebrates [1]–[3]. For example, in the fly wing the core proteins ensure that the single trichome that emerges from each cell always points towards the distal end of the wing. Furthermore, in the eye core proteins regulate the orientation and chirality of photoreceptor clusters (ommatidia). The core proteins localise asymmetrically at proximal and distal cell ends in the wing, or at the R3/R4 photoreceptor cell boundary in the eye. In the wing, Prickle localises proximally, together with the transmembrane proteins Strabismus (Stbm, also known as Van Gogh [Vang]) and Flamingo (Fmi, also known as Starry Night [Stan]), whilst Fmi also localises to distal cell ends together with Frizzled (Fz), Dishevelled (Dsh) and Diego (Dgo).

Loss of any single core protein disrupts the asymmetric localisation of the others. Fz, Fmi and Stbm appear to assemble into an intrinsically asymmetric intercellular complex that couples adjacent cells, and Pk and the other cytoplasmic core proteins (Dsh and Dgo) are then thought to organise intercellular complexes of the same polarity into discrete membrane domains at the proximal and distal cell edges [4]. This redistribution can be explained by feedback models, consisting of either positive (stabilising) interactions between complexes in the same orientation or negative (destabilising) interactions between complexes in opposite orientations [4]–[6].

As asymmetric complexes span cell boundaries, feedback amplification would be sufficient to locally coordinate polarity between neighbouring cells, but not necessarily sufficient to align this with the axes of the tissue [7]–[9]. Thus it is widely believed that upstream cues provide a weak polarising bias to each cell, which is then coordinately amplified to give robust asymmetry. The nature of these upstream cues is controversial, although in some contexts it appears to involve gradients of activity of the atypical cadherins Fat (Ft) and Dachsous (Ds) (reviewed in [10]–[12]).

The *pk* gene has three splice forms that give rise to three isoforms of the protein product, Pk^Pk^, Pk^Sple^ (hereafter Pk and Sple) and Pk^M^. Pk and Sple differ in that Sple has a longer N-terminal extension, whilst Pk^M^ is only expressed in the embryo and has no known function [13]. Loss of both Pk and Sple isoforms (*pk^pk-sple^* mutants) results in adult phenotypes similar to those seen upon loss of the other core proteins: hairs on the wing swirl in a characteristic pattern as a result of trichomes forming in the centre of cells in which the core proteins no longer exhibit noticeable asymmetric localisation [7], [14], [15]. Similarly in the eye, ommatidia adopt random chiralities and misrotate [16], and tarsal joint duplications are seen in the leg [13].

In contrast, loss of only the Pk splice form (*pk^pk^* flies) does not affect the eye or leg, but a strong polarity phenotype is seen in the wing whereby trichomes point towards vein 3 [14]. Conversely, loss of the Sple splice form (*pk^sple^* flies) does not affect the wing, but ommatidia in the eye adopt random chirality and there are tarsal joint duplications in the leg [13], [16]. Furthermore, overexpression of Sple in the wing gives a reversal of trichome polarity which is similar to but more extreme than the *pk^pk^* phenotype, whilst overexpression of Pk in the leg gives strong joint duplications [13]. It has been suggested that the *pk^pk^* and *pk^sple^* mutant phenotypes are due to specific roles for the two isoforms in interpreting global cues in different tissues [17], [18]. In particular, gradients of Ft/Ds activity have been proposed to orient core protein complexes containing Sple, but to have little influence on Pk-containing complexes [19].

We have recently presented evidence that in order for feedback amplification to occur correctly, the levels of core proteins at junctions must be tightly regulated [20]. For example an excess of core proteins might disrupt negative interactions by excluding too much of a competitor protein from a membrane domain, or disrupt positive interactions by causing excessive stabilisation of complexes that then spread into inappropriate domains. In support of this, we found that neddylation and ubiquitination control the levels of Dsh at junctions and that this is required for optimal polarisation [20]. Neddylation is the covalent attachment of the small ubiquitin-like molecule Nedd8 to target proteins, which can alter protein activity or stability, with Cullin (Cul) E3 ubiquitin ligase subunits being the best understood targets [21]. In the wing, neddylation regulates a Cul-3-Diablo/Kelch E3 ubiquitin ligase which acts to remove excess Dsh from junctions, and loss of this activity results in an increase in Dsh levels. This promotes the accumulation of all the other core proteins and results in reduced core protein asymmetry [20]. Interestingly, overexpression of Dsh, Pk and Dgo all cause accumulation of the other core proteins at junctions [5], [22], [23], leading to the possibility that comparable mechanisms also control the levels of Pk and Dgo. No such mechanisms have been identified in flies, although levels of vertebrate Pk are regulated by a Smurf ubiquitin ligase [24].

Pk is localised to proximal cell edges with Stbm, and as the two proteins interact *in vitro* [6], [23], it is thought that Pk is recruited to junctions by Stbm. However, Pk has a prenylation motif at its C-terminus (CaaX, where cysteine is the site of prenylation, a is an aliphatic residue and X determines the type of prenyl group added). Prenylation can take the form of addition of either a farnesyl or geranylgeranyl moiety, and normally acts to allow association of cytoplasmic proteins with cell membranes, although a second signal is often needed for stable membrane association [25]. If Pk is normally recruited to the plasma membrane by Stbm an additional need for it to be prenylated is unclear, although it could stabilise a weak interaction with Stbm [26], [27]. Previous studies have indicated that loss of the prenylation motif may reduce the association of Pk with junctions [28], [29], and that some Pk phenotypes can be phenocopied with a farnesylation inhibitor [30], but other experiments suggested that prenylation is not essential for Pk function [6], and it has yet to be established whether Pk is indeed prenylated *in vivo*.

Here, we demonstrate that Pk is turned over rapidly in pupal wing cells, and that this turnover is dependent on Stbm activity. Furthermore, we show that Pk is farnesylated *in vivo*, and that farnesylation of Pk promotes its recruitment to junctions by Stbm, where it participates in feedback amplification. Additionally, this recruitment is also necessary for Stbm to promote degradation of excess Pk.

## Results

### Pk degradation is regulated by Stbm

**Figure 1:**
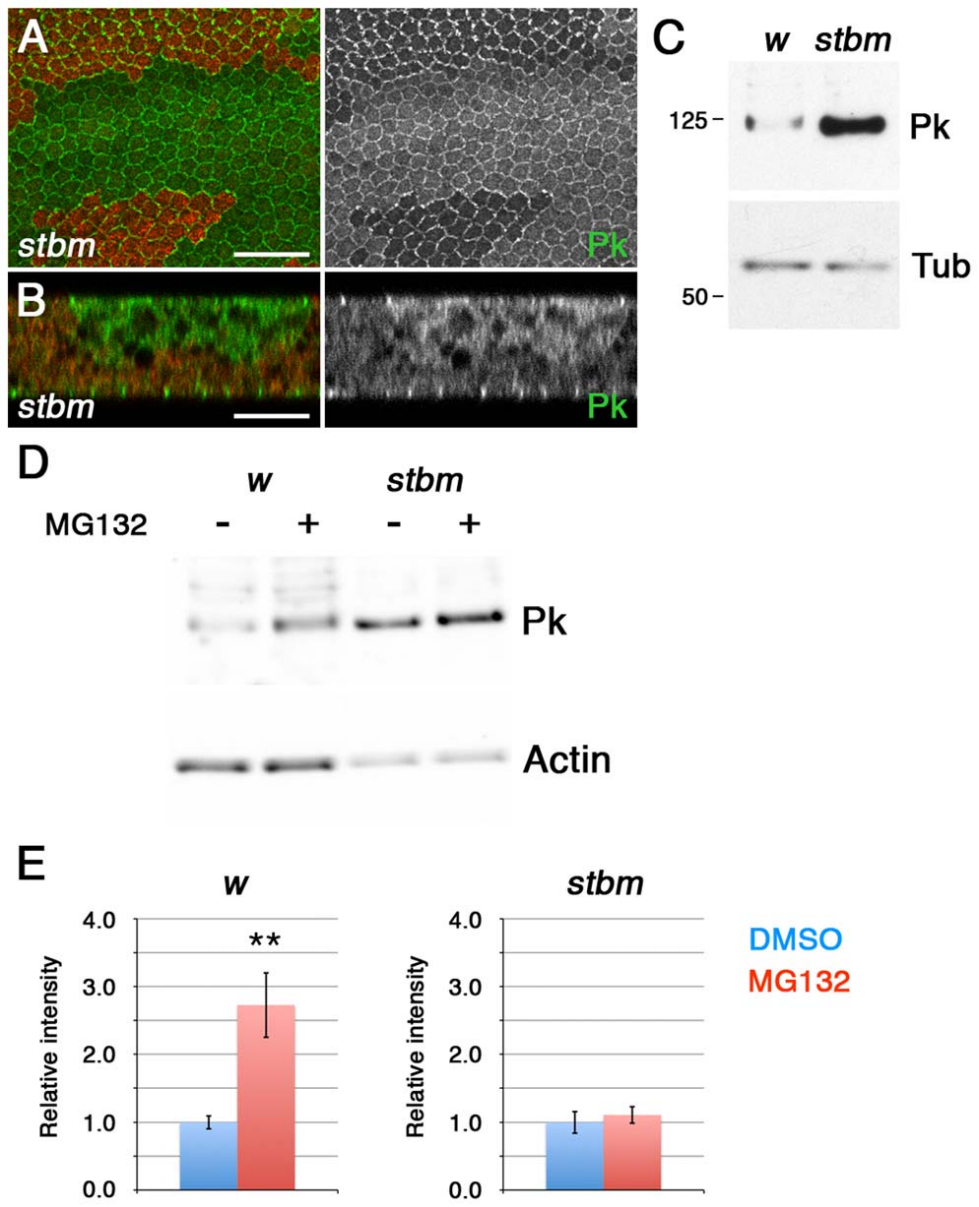
Stbm regulates Pk localisation and degradation. (A,B) XY (A) or XZ (B) sections of Pk staining (green) in *stbm^6^* clones, marked by loss of ß-gal staining (red). The XZ section shows the two apposed epithelia of the pupal wing, and the clone is only present in the top epithelium. Scale bar 20 µm (A) or 10 µm (B). (C) Western blot probed with anti-Pk showing Pk levels in *w^1118^* and *stbm^6^* 28 hr pupal wings, with α-Tubulin (Tub) as loading control. Pk levels are approximately 20-fold increased in *stbm^6^* wings. (D,E) Western blot (D) and quantitation (E) of Pk levels relative to Actin levels, in *w^1118^* or *stbm^6^* prepupal wings treated with 10 µM MG132, or DMSO control, for 5 hr at 25°C. Quantitation from 4 biological replicates, error bars are s.e.m., **p = 0.01.

We and others have previously shown that loss of Stbm activity causes Pk to become more cytoplasmic (Figure 1A, [6], [23]), consistent with Stbm recruiting Pk to junctions. Interestingly, Pk did not seem to be merely redistributed from junctions to the cytoplasm, but overall levels of Pk also appeared to increase (Figure 1B). This was confirmed by Western blot analysis of total Pk levels in wild type and *stbm* pupal wings (Figure 1C). The increase in Pk levels was not due to increased transcription of *pk*, as levels of EGFP-Pk expressed under control of the *Actin5C* promoter also increased in *stbm* mutants (Figure S1A,B). Therefore, Stbm both recruits Pk to junctions and regulates its levels.

To test whether Stbm regulates Pk levels by promoting its degradation, we investigated Pk turnover in prepupal wings. Interestingly, treatment of prepupal wings with MG132 to block proteasomal degradation caused a substantial increase in Pk levels (Figure 1D,E), consistent with Pk normally being rapidly degraded in the proteasome. Importantly, if *stbm* mutant wings were treated with MG132, there was no additional increase in Pk levels (Figure 1D,E), suggesting that Stbm is necessary for the proteasomal degradation of Pk. No accumulation of Pk was seen if lysosomal degradation was blocked (Figure S1C), confirming that degradation is through the proteasome rather than the lysosome.

### Pk is farnesylated *in vivo*

We were interested in what else might influence Pk recruitment by Stbm and its degradation. One possibility is that if Pk were prenylated (by addition of either a farnesyl or geranylgeranyl group) this could target it to membranes, and promote or accelerate the interaction of Pk with Stbm. Previous analyses of the requirement for Pk prenylation in flies have variously concluded that the prenylation motif was not essential for Sple function [6], or alternatively that it might be required for correct localisation of Pk, but partially dispensable for localisation of Sple [28]. However, as these experiments only looked at one Pk isoform, or relied on overexpression to assay the effect of loss of the prenylation motif, we decided to re-examine this issue.

**Figure 2:**
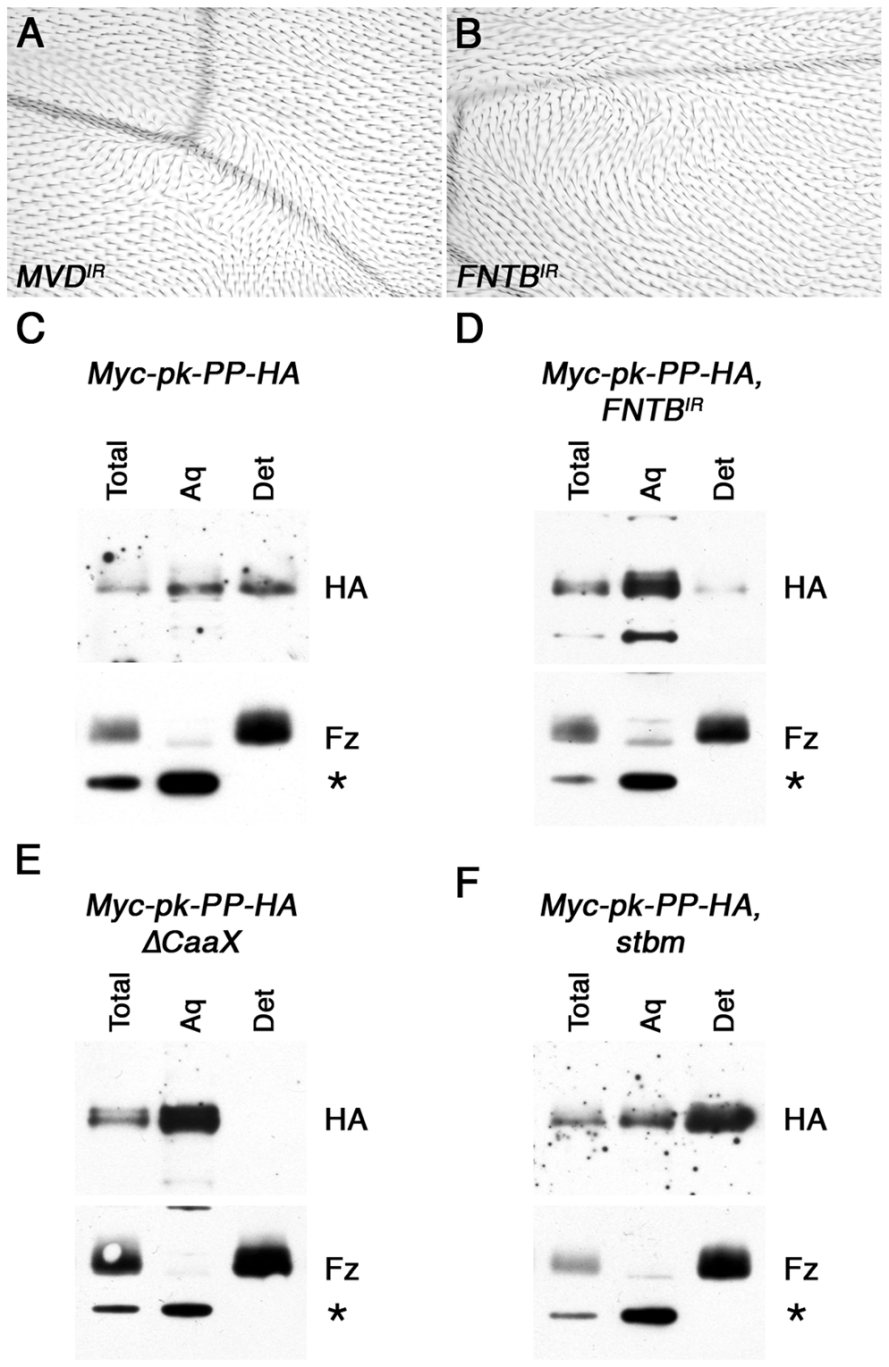
*In vivo* farnesylation of Pk. (A,B) Adult wings from *MS1096-GAL4, MVD^IR-24253^* (A) and *459.2-GAL4, FNTB^IR-17565R-2^* (B) flies. (C–F) Western blots showing phase separation of the HA-tagged C-terminus of Myc-Pk-PP-HA, after cleavage with Prescission protease. Blots show HA staining or control Fz staining (bottom) of 28 hr pupal wing extracts from *ActP-Myc-pk-PP-HA/+* (C), *MS1096-GAL4; FNTB^IR-17565R-2^/+; ActP-Myc-pk-PP-HA/+* (D), *ActP-Myc-pk-PP-HAΔCaaX/+* (E) or *stbm^6^; ActP-Myc-pk-PP-HA/+* (F), with total lysate, aqueous fraction (Aq) or detergent fraction (Det). Fz partitions into the detergent phase, and a cross-reacting band partitions only in the aqueous phase (asterisk).

We have recently performed an RNAi screen in the adult wing, in which 10,000 RNAi lines were expressed using the *MS1096-GAL4* driver (H.S, V.T.-M., C. Thomas and D.S., unpublished data). This identified two genes encoding components of the HMG CoA Pathway, which when knocked down caused trichomes to swirl (Figure 2A,B, Table S1). The HMG CoA pathway is the biosynthetic pathway that leads to formation of farnesyl and geranylgeranyl isoprenoids, which are then covalently attached to cysteine residues near the C-terminus of target proteins (Figure S2A, [25]). Of the components identified in our screen, CG8239 encodes diphosphomevalonate decarboxylase (MVD), which is required for both farnesyl and geranylgeranyl synthesis, and CG17565 encodes one of the two subunits of farnesyl-diphosphate farnesyl transferase (FNTB), consistent with the possibility that Pk is normally farnesylated.

To see whether Pk is a target of MVD and FNTB, we tested directly if Pk is prenylated *in vivo*, using a phase extraction technique that is commonly used to assess prenylation of other proteins such as small GTPases. In this assay, proteins are extracted using the detergent Triton X-114, which is fully miscible with aqueous solutions at 4°C, but separates into aqueous and detergent phases above 20°C [31], such that transmembrane proteins and prenylated proteins are partitioned into the detergent phase. We first carried out this assay on endogenous Pk protein; however no prenylation of Pk was detected (data not shown), possibly due to the Pk protein being several-fold larger than proteins normally used in this assay, and thus not being efficiently partitioned into the detergent phase by a small hydrophobic farnesyl tag.

To circumvent this, we generated an engineered form of Pk that is tagged at the N-terminus with Myc, and has a cleavage site for Prescission protease (PP) followed by a HA tag within a non-conserved region near its C-terminus (Figure S2B). This protein was expressed in flies under control of the *Actin5C* promoter, and was seen to localise asymmetrically in pupal wings and to fully rescue *pk^pk-sple13^* mutant wings (Figure S2C,D). As expected, no cleavage at the PP cleavage site was observed *in vivo*; however addition of PP to pupal wing extracts led to efficient cleavage, and the release of a small HA-tagged C-terminal fragment of Pk (Figure S2E), which could be tested for prior *in vivo* farnesylation using phase extraction.

Using this methodology, the cleaved C-terminus of Myc-Pk-PP-HA protein was seen to partition in both the aqueous and detergent fractions, whereas a control blot for the transmembrane protein Fz showed it partitioning exclusively in the detergent fraction, and a cross-reacting band was exclusively cytoplasmic (Figure 2C). This suggests that a substantial proportion of Pk is farnesylated *in vivo*. This observation was confirmed in two ways. Firstly, Myc-Pk-PP-HA was expressed in wings in which *FNTB* was knocked down: in this case the HA-tagged C-terminus of Pk partitioned almost entirely in the aqueous phase (Figure 2D). Secondly, expression of a protein in which the C-terminal prenylation motif was deleted (Myc-Pk-PP-HAΔCaaX) resulted in its partitioning only to the aqueous phase (Figure 2E).

### Pk farnesylation is essential for its function in the wing

**Figure 3:**
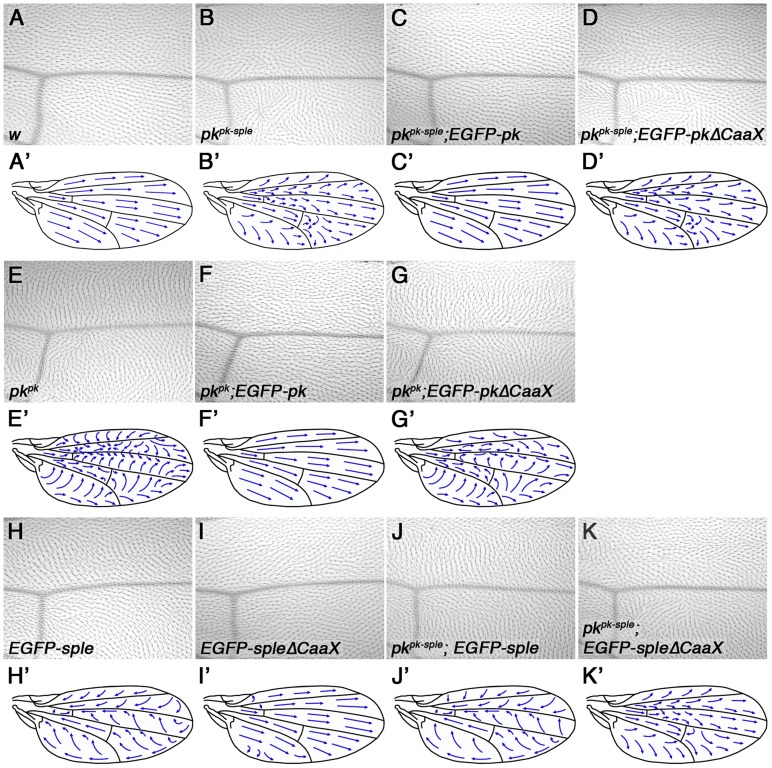
Requirement for Pk/Sple farnesylation in the wing. (A–K) Images of the region around vein 3 distal to the posterior cross-vein (A–K) and cartoons (A'–K') of the dorsal surface of adult wings from wild type (A), *pk^pk-sple13^* (B), *pk^pk-sple13^*; *ActP*-*EGFP-pk/+* (C), *ActP*-*EGFP-pkΔCaaX/+; pk^pk-sple13^* (D), *pk^pk1^* (E), *pk^pk1^*; *ActP*-*EGFP-pk/+* (F), *ActP*-*EGFP-pkΔCaaX/+; pk^pk1^* (G), *ActP-EGFP-sple/+* (H), *ActP-EGFP-spleΔCaaX/+* (I), *pk^pk-sple13^*; *ActP*-*EGFP-sple/+* (J) and *pk^pk-sple13^*; *ActP*-*EGFP-spleΔCaaX/+* (K) flies. Mild swirls are present in the proximal regions of *ActP-EGFP-spleΔCaaX* wings.

We then tested whether farnesylation of Pk was necessary for its function. EGFP-tagged Pk or Sple were expressed under control of the *Actin5C* promoter, as either full-length forms or forms lacking the prenylation motif (ΔCaaX). Whilst EGFP-Pk fully rescued *pk^pk-sple^* and *pk^pk^* wings (Figure 3C,F, compare to Figure 3B,E), EGFP-PkΔCaaX did not show significant rescue (Figure 3D,G). Therefore we conclude that farnesylation is required for Pk activity in the wing.

**Figure 4:**
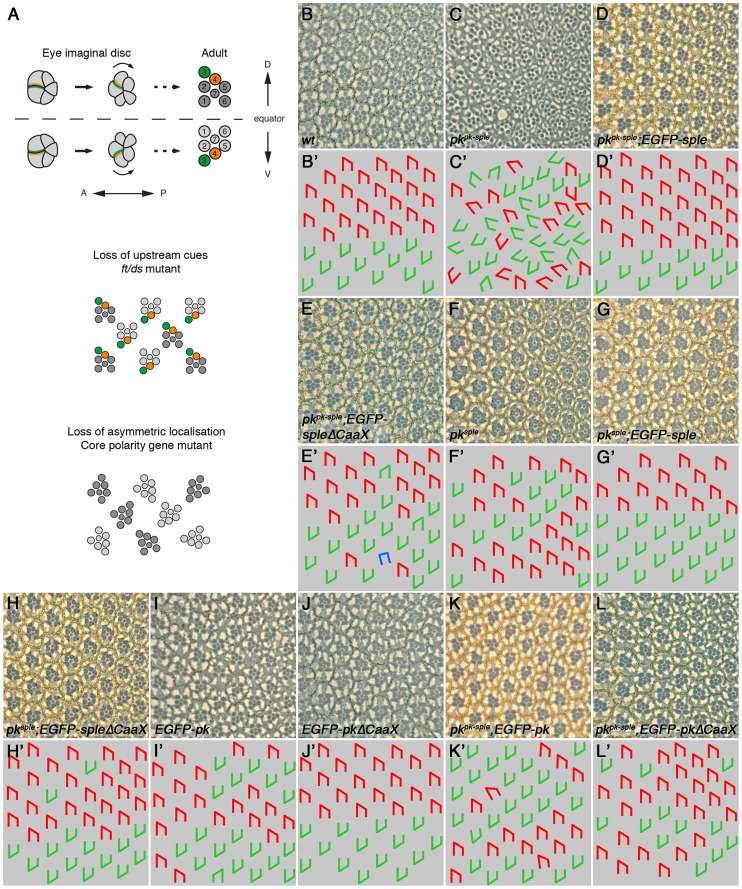
Partial requirement for Pk/Sple farnesylation in the eye. (A) Cartoon showing ommatidial polarity and rotation. In the eye imaginal disc, photoreceptor cells are recruited in a wave from posterior (P) to anterior (A). Photoreceptor cell clusters are initially symmetric, but Fz (green) becomes localised to the R3/R4 cell boundary, in the cell closest to the dorsal-ventral (DV) midline, or equator. Stbm/Sple (orange) localise to the apposing cell edge. This specifies R3/R4 cell fate, and causes ommatidia to adopt opposite chirality on either side of the equator, and to rotate 90° in opposite directions. In eyes in which upstream cues have been lost, core proteins localise asymmetrically and rotate 90° as normal, but the R3/R4 fate decision is randomised and ommatidia adopt random chirality. In core polarity gene mutants, asymmetric localisation of the other core proteins is lost or delayed, and ommatidia adopt random chirality and rotate to a random degree. (B–L) Adult eye sections (B–L), and cartoons (B'–L'), of a region around the equator from wild type (B), *pk^pk-sple13^* (C), *pk^pk-sple13^*; *ActP*-*EGFP-sple/+* (D), *pk^pk-sple13^*; *ActP*-*EGFP-spleΔCaaX/+* (E), *pk^sple1^* (F), *pk^sple1^*; *ActP*-*EGFP-sple/+* (G), *pk^sple1^*; *ActP*-*EGFP-spleΔCaaX/+* (H), *ActP-EGFP-pk/+* (I), *ActP-EGFP-pkΔCaaX/+* (J), *pk^pk-sple13^*; *ActP*-*EGFP-pk/+* (K) and *ActP*-*EGFP-pkΔCaaX/+; pk^pk-sple13^* (L) flies. *pk^pk-sple^* and *pk^sple^* eyes are completely rescued by *ActP*-*EGFP-sple*. 9% and 5% of ommatidia are still inverted for *pk^pk-sple13^*; *ActP*-*EGFP-spleΔCaaX/+* and *pk^sple1^*; *ActP-EGFP-spleΔCaaX*, respectively, but the misrotation phenotype is completely rescued. 1% of ommatidia are misrotated in *pk^pk-sple13^*; *ActP*-*EGFP-pk/+* and *ActP*-*EGFP-pkΔCaaX/+; pk^pk-sple13^* eyes.

Similarly, we saw complete rescue of *pk^pk-sple^* and *pk^sple^* eyes and legs using EGFP-Sple (Figure 4D,G, compare to Figure 4C,F, and Figure S3B,C,D,F). Interestingly, EGFP-SpleΔCaaX also gave substantial (but not complete) rescue in both cases (Figures 4E,H,S3E,G). Therefore we conclude, in agreement with earlier findings in the eye [6], that farnesylation is only partially required for Sple function in the eye and leg.

These differing results could indicate that farnesylation is more important for Pk/Sple function in the wing than in the eye/leg, or might indicate that Pk has a more critical requirement for farnesylation than Sple (regardless of the tissue in which they are active). To distinguish between these possibilities, we expressed EGFP-Sple and EGFP-SpleΔCaaX in the wing. When expressed under the *Actin5C* promoter, EGFP-Sple caused a dominant *pk^pk^*-like phenotype, with trichomes pointing proximally and towards vein 3 (Figure 3H). Under these conditions, EGFP-Sple localised asymmetrically, at cell edges opposite to the site of trichome initiation (Figure S4A). A similar *pk^pk^*-like phenotype was seen when EGFP-Sple was expressed in a *pk^pk-sple^* mutant background (Figure 3J). However, EGFP-SpleΔCaaX did not localise asymmetrically (Figure S4B), did not cause a dominant phenotype (Figure 3I) and did not alter the trichome polarity phenotype of a *pk^pk-sple^* mutant (Figure 3K). Thus, EGFP-SpleΔCaaX is unable to substitute for EGFP-Sple in the wing.

In the converse experiment, expression of EGFP-Pk, but not EGFP-PkΔCaaX was able to give a dominant *pk^sple^*-like phenotype in the eye (Figure 4I,J). However, both EGFP-Pk and EGFP-PkΔCaaX rescued the misrotation (but not chirality) phenotype of *pk^pk-sple^* eyes (Figure 4K,L). Therefore, EGFP-PkΔCaaX is able to partially substitute for EGFP-Pk in the eye.

We conclude from this that the wing is more sensitive than the eye to loss of farnesylation activity, regardless of which isoform is used. In the wing, we find that farnesylation is required for either the Pk or Sple isoforms to participate in asymmetric complex formation and for controlling alignment of asymmetric complexes with the tissue axes. In the eye, farnesylation of Pk or Sple appears partially dispensable for ommatidial rotation (which depends on asymmetric complex formation), and farnesylation of Sple is also largely dispensable for determination of ommatidial chirality (a measure of correct coupling to the tissue axes). However, misexpression of Pk in the eye reveals an absolute requirement for farnesylation of Pk for disrupting ommatidial chirality and thus normal coupling to the tissue axes (see Discussion).

### Loss of farnesylation causes an increase in cytoplasmic levels of Pk

**Figure 5:**
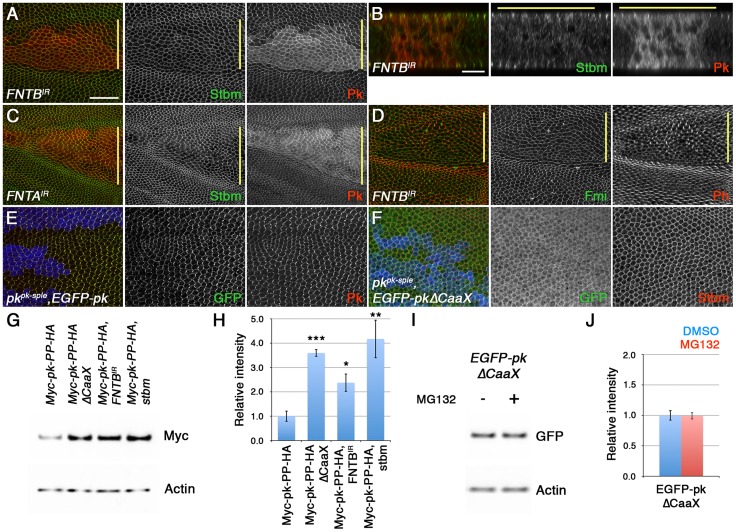
Loss of farnesylation causes an increase in cytoplasmic Pk. (A–D) XY (A,C,D) and XZ (B) sections of pupal wings expressing *ptc-GAL4/FNTB^IR-17565R-2^; UAS-Dcr2/+* (A,B,D) or *ptc-GAL4/+; FNTA^IR-2976R-4^/UAS-Dcr2* (C). In A-C, staining is for Stbm (green) and Pk (red), and in D staining is for Fmi (green) and Phalloidin (red). (E,F) *pk^pk-sple13^* clones, marked by loss of ß-gal staining (blue), in wings expressing *ActP-EGFP-pk* (E) or *ActP-EGFP-pkΔCaaX* (F). Staining is for GFP (green) and Pk (red in E) or Stbm (red in F). Yellow bar marks the *ptc-GAL4* domain. Scale bar 20 µm (A,C–F) or 10 µm (B). (G,H) Western blot (G) and quantitation (H) of Myc levels relative to Actin levels, in 28 hr pupal wings extracts from *ActP-Myc-pk-PP-HA/+*, *ActP-Myc-pk-PP-HAΔCaaX/+*, *MS1096-GAL4/w; FNTB^IR-17565R-2^/+; ActP-Myc-pk-PP-HA/+* and *stbm^6^; ActP-Myc-pk-PP-HA/+* flies. Quantitation from 3 biological replicates, error bars are s.e.m., p***<0.001, p**<0.01, p*<0.05. (I,J) Western blot (I) and quantitation (J) of GFP levels relative to Actin levels, in extracts from *ActP-EGFP-pkΔCaaX* prepupal wings treated with 10 µM MG132, or DMSO control, for 5 hr at 25°C. Quantitation from 2 biological replicates, error bars are s.e.m.

Expression of RNAi targeting the two farnesyl transferase subunits (FNTA and FNTB) in pupal wings resulted in a disruption in core protein asymmetry (Figure 5A,C) and trichome polarity (Figures 2B and 5D). Notably, Pk also became more cytoplasmic, and overall levels appeared to increase (Figure 5A,B,C). Furthermore, whilst EGFP-Pk expressed under the *Actin5C* promoter localised strongly to junctions and was distributed asymmetrically (Figure 5E), EGFP-PkΔCaaX was more cytoplasmic, and no asymmetry of the remaining junctional population could be detected (Figure 5F). Similar effects were seen for EGFP-Sple and EGFP-SpleCaaX (Figure S4A,B), although EGFP-SpleΔCaaX appeared more junctionally localised than EGFP-PkΔCaaX (compare Figures S4B and 5F).

We then investigated if loss of farnesylation did indeed lead to an increase in total Pk levels. Our *Actin-EGFP-pk* and *Actin-EGFP-pkΔCaaX* transgenes were not inserted into the same genomic location, so although levels of EGFP-PkΔCaaX were higher (Figure S4D), we could not exclude the possibility that this was due to greater transcription of *EGFP-pkΔCaaX*. However, the *Actin-Myc-pk-PP-HA* and *Actin-Myc-pk-PP-HAΔCaaX* transgenes used for the phase extraction experiments are inserted into the same genomic location and should thus be expressed at equivalent levels. Notably, there was three-fold more Myc-Pk-PP-HAΔCaaX protein in pupal wings than Myc-Pk-PP-HA protein, similar to the amount of Myc-Pk-PP-HA protein detected in a *stbm* mutant (Figure 5G,H). Furthermore, in wings in which FNTB was knocked down, Myc-Pk-PP-HA levels also increased (Figure 5G,H).

Finally, if the increase in levels of Pk that cannot be farnesylated is due to it no longer being degraded, we would expect that blocking proteasomal degradation would not cause any further increase in Pk levels. Indeed, EGFP-PkΔCaaX levels did not increase after MG132 treatment (Figure 5I,J). We conclude that non-farnesylated Pk escapes proteasomal degradation.

### Farnesylation is necessary for Pk to be recruited and degraded by Stbm

In *stbm* mutants, or when Pk cannot be farnesylated, we see the same phenotype: a failure in recruitment of Pk to junctions, and a failure in Pk degradation. One possibility is that Stbm could be required for Pk farnesylation, and in the absence of farnesylation Pk accumulates in the cytoplasm. Alternatively, farnesylation could be required for Pk to interact with Stbm, and in the absence of this interaction, Stbm is unable to promote the degradation of Pk.

We first examined whether Stbm was required to farnesylate Pk. Significantly, phase extraction showed that Myc-Pk-PP-HA was still farnesylated in the absence of *stbm* (Figure 2F), indicating that this was not the case. Furthermore, we failed to detect farnesylation of Pk in tissue culture cells regardless of whether Stbm was cotransfected or not (Figure S5A).

**Figure 6:**
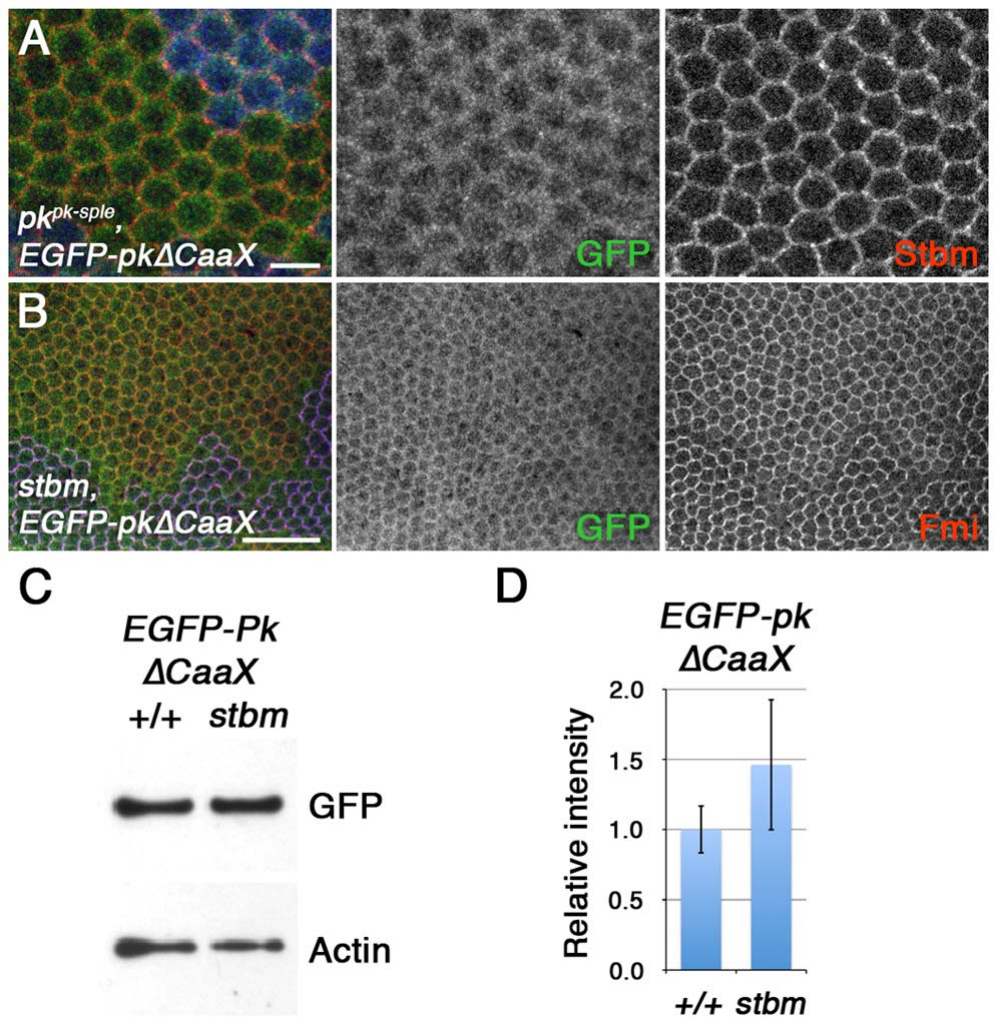
Farnesylation is required for Stbm to promote Pk recruitment to junctions and Pk degradation. (A) High magnification image of *pk^pk-sple13^* pupal wing clone, marked by loss of ß-gal staining (blue), in wings expressing *ActP-EGFP-pkΔCaaX*. Staining is for GFP (green) and Stbm (red). Scale bar 5 µm. (B) *stbm^6^* pupal wing clone, marked by loss of Stbm (blue) staining, in wings expressing *ActP-EGFP-pkΔCaaX*. Staining is for GFP (green) and Fmi (red). Scale bar 20 µm. (C,D) Western blot (C) and quantitation (D) of GFP levels relative to Actin levels, in 28 hr pupal wing extracts from *ActP-pkΔCaaX/+* and *pActP-pkΔCaaX/+; stbm^6^* flies. Quantitation from 3 biological replicates, error bars are s.e.m., p = 0.40.

We next examined if Pk farnesylation was required for Stbm to bind to Pk. In tissue culture cells, both full-length Pk or PkΔCaaX could co-immunoprecipitate Myc-tagged Stbm, suggesting that farnesylation is not an absolute requirement for Stbm to interact with Pk (Figure S5B). However, high magnification imaging of pupal wings showed that whilst some unfarnesylated Pk localised in the vicinity of junctions, staining was quite diffuse and the co-localisation of Pk with Stbm was very poor (Figure 6A). Furthermore, EGFP-PkΔCaaX localisation was not dependent on Stbm activity, as it was not noticeably altered in a *stbm* mutant (Figure 6B). Junctional localisation was also not dependent on endogenous Pk, as again there is little alteration in EGFP-PkΔCaaX localisation in a *pk^pk-sple^ stbm* double mutant (Figure S4C). Finally, overall levels of EGFP-PkΔCaaX did not alter in a *stbm* mutant (Figure 6C,D).

Overall, this supports the view that although Stbm may be capable of binding unfarnesylated Pk *in vitro*, this binding is insufficient for Stbm to recruit Pk into asymmetric complexes, and to promote degradation of excess Pk *in vivo*.

### Regulation of Pk degradation via the SkpA SCF ubiquitin ligase subunit

We have previously shown that loss of the Nedd8 conjugating enzyme Ubc12 increases Dsh levels at junctions [20]. Loss of neddylation modulates activity of a Cul-3 ubiquitin ligase complex, which leads to increased levels of Dsh, and thus other core proteins, at junctions. Interestingly, there also seems to be a second target for neddylation, independent of Cul-3 and Dsh, as loss of Dsh activity does not completely abolish the increase in levels of the other core proteins seen in *Ubc12* mutant wings [20].

**Figure 7:**
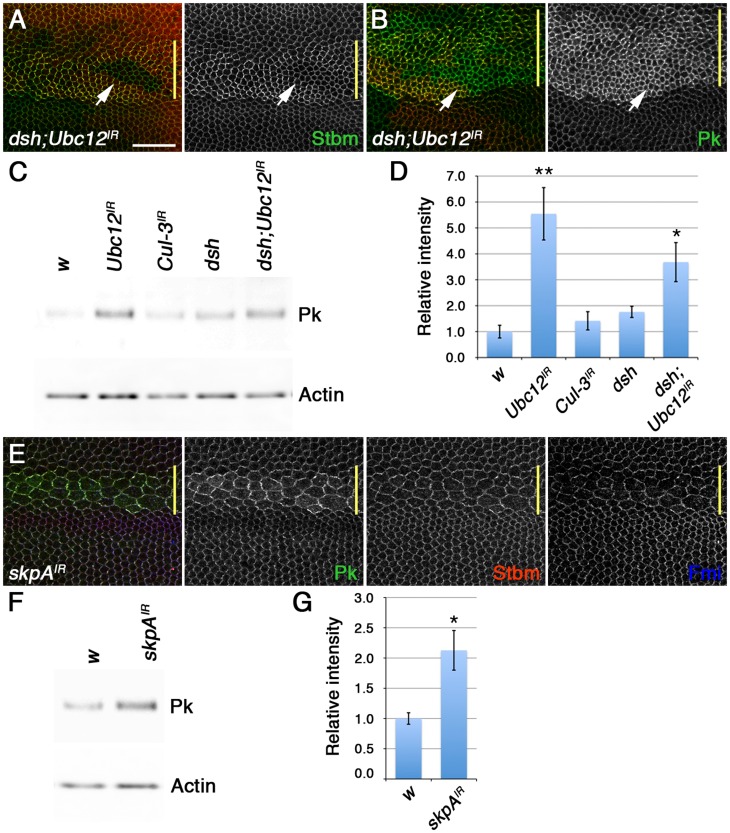
The SkpA ubiquitin ligase subunit regulates Pk degradation. (A,B) *dsh^V26^* clones, marked by loss of Dsh staining (red), in wings expressing *ptc-GAL4/+; Ubc12^IR-7375R-3^/+*, stained for Stbm (green in A) or Pk (green in B). Note that Pk still accumulates at high levels at junctions when *Ubc12* RNAi is expressed in *dsh^V26^* clones, but Stbm does not (arrows). Yellow bar marks the *ptc-GAL4* domain. Scale bar 20 µm. (C,D) Western blot (C) and quantitation (D) of Pk levels relative to Actin levels, in 28 hr pupal wing extracts from *w^1118^*, *MS1096-GAL4; Ubc12^IR-7375R-3^/+*, *MS1096-GAL4; Cul3^IR-109415^/+*, *dsh^1^* and *dsh^1^ MS1096-GAL4; Ubc12^IR-7375R-3^* male flies. Quantitation from 3 biological replicates. p*<0.05, p**<0.01. (E) Pk (green), Stbm (red) and Fmi (blue) staining of wings expressing *ptc-GAL4/skpA^IR-46605^*. Note that whilst the increase in Pk levels in wings expressing *Ubc12* RNAi may contribute to clustering of the other core proteins, the increase in Pk in wings expressing low levels of *skpA* RNAi is insufficient to do this. (F,G) Western blot (F) and quantitation (G) of Pk levels relative to Actin levels, in 28 hr pupal wing extracts from *w^1118^* and *MS1096-GAL4/+; skpA^IR-46605^/+* female flies. Quantitation from 3 biological replicates, error bars are s.e.m,. p*<0.05.

A number of lines of evidence suggests that this second target could be Pk. Firstly, levels of Pk were still elevated in *dsh* clones after *Ubc12* knockdown, whilst levels of other core proteins were largely rescued (Figure 7A,B). This suggests that Pk levels increase non-stoichiometrically with respect to Stbm. Furthermore, a strong increase in total Pk levels was observed in wings in which *Ubc12* was uniformly knocked down (Figure 7C,D). This is not a secondary consequence of increased Dsh levels, as no corresponding increase in Pk levels was seen when *Cul-3* was knocked down, and Pk levels still increased when *Ubc12* was knocked down in a *dsh^1^* mutant background (Figure 7C,D).

We then postulated that the neddylation pathway might act on Pk indirectly by neddylating another Cullin. In our previous work we identified the Cul acting on Dsh by analysing total Fmi levels, but did not examine Pk levels. Therefore, we screened RNAi lines targeting the remaining 4 *Drosophila* Cul proteins, looking for changes in Pk staining. No evident increase in Pk levels at junctions was seen when RNAi against *Cul-2*, *Cul-4* and *Cul-5* was expressed in the pupal wing, and RNAi against *lin19/Cul-1* caused larval lethality (data not shown). However, RNAi targeting *skpA*, which encodes a subunit of an SCF (Skp1/Cullin-1/F-Box) E3 ubiquitin ligase, was not lethal when expressed at low temperatures, although there was substantial disruption of cells within the expression domain. Nevertheless, elevated levels of Pk were seen in cells expressing the RNAi (Figure 7E). The specificity of this effect was confirmed using an independent short homologous RNAi line (Figure S6A). Furthermore, *skpA* knockdown caused an increase in the cytoplasmic levels of Armadillo, a known Cul-1 target (Figure S6B). Notably, total Pk levels also increased in wings in which *skpA* was uniformly knocked down (Figure 7F,G). Therefore, we propose that the interaction of Pk with Stbm at membranes promotes proteolytic degradation of Pk via a Cul-1 dependent mechanism.

## Discussion

### Stbm regulates junctional recruitment and degradation of farnesylated Pk

We find that whilst Stbm is required for recruitment of Pk into junctional complexes [6], [23], it also promotes Pk degradation. One possibility is that if Pk forms asymmetric complexes with Stbm and other core proteins, it is protected from degradation, but if Pk is localised to the plasma membrane without entering an asymmetric complex then Stbm triggers its degradation. If Pk functions in feedback loops, this might act as a mechanism to restrict Pk action to cellular sites where Stbm is in asymmetric complexes. Notably, we recently reported a similar mechanism involving Dsh, whereby a population of Dsh at junctions is subject to degradation mediated by a Cullin-3/Diablo/Kelch E3 ubiquitin ligase [20]. Therefore, this could be a general mechanism for limiting the amount or activity of the cytoplasmic core proteins operating in feedback loops.

We note that there seem to be differences in the ability of the cytoplasmic proteins, when in excess, to stabilise the other core proteins at junctions. Excess Dsh at junctions caused by loss of Cul-3 or Dbo/Kel activity results in a striking accumulation of the other core proteins [20], whilst there is only a mild increase in the case of excess Pk (for example when *Ubc12* activity is knocked down in a *dsh* background, Figure 7A). This may suggest that Dsh is better at stabilising the other core proteins than Pk, and is consistent with the observation that loss of Dsh has stronger effects on amplification of asymmetry [32].

How Pk might be targeted for degradation is unknown, but degradation is dependent on the SCF complex component skpA and the proteasome. It is unclear whether Pk is a direct target of an SCF complex. Interestingly, in vertebrates a Smurf ubiquitin ligase was demonstrated to target Pk for degradation [24]; however Smurf is a HECT E3 ubiquitin ligase and thus does not act in a complex with Cullins. Furthermore, no planar polarity defects were seen when we expressed RNAi against the fly Smurf homologue, although the extent of Smurf knockdown was not assessed (E. Searle and D.S., unpublished data).

We also show for the first time that Pk is farnesylated *in vivo*, and that farnesylation of Pk is a prerequisite for stable localisation of Pk with Stbm, and for it to function in asymmetric complex formation and clustering of core proteins into junctional puncta. Furthermore, farnesylation is also necessary for Stbm to control Pk levels, consistent with Stbm triggering degradation of Pk that is already in membranes.

Whether Pk localisation to junctions specifically requires farnesylation, or whether another lipid modification could be substituted, is unknown. Nevertheless, the chances of a cytoplasmic protein meeting a transmembrane protein are much lower than the chances of two transmembrane proteins meeting [33]. Therefore, we propose that the role of farnesylation is to promote Pk localisation to membranes, where it is more likely to interact with Stbm. Hence farnesylation is required both for Stbm-Pk containing asymmetric complexes to form, by synergising with weak direct interactions between Stbm and Pk, and also for Stbm to promote degradation of excess Pk.

### Differential requirements for Pk and Sple farnesylation in the wing and eye

Farnesylation is essential for Pk/Sple function in the wing, but appears to be less important in the eye and leg. In the case of Sple, the apparent reduced requirement for farnesylation for its activity in the eye might have been due to its unique N-terminus bypassing the need for farnesylation. Interestingly, EGFP-SpleΔCaaX does appear to localise better to junctions in the wing than EGFP-PkΔCaaX (compare Figures 5F and S4B). However, EGFP-SpleΔCaaX does not localise asymmetrically in the wing (Figure S4B), nor can it rescue *pk^pk-sple^* mutants (Figure 3K), suggesting its ability to partially rescue in the eye cannot be explained simply by it associating more strongly to junctions.

An alternative explanation for the ability of non-farnesylated Sple to partially function in the eye but not the wing is simply that less Sple activity is necessary for the R3/R4 fate decision than for trichome placement. In the eye, the core proteins localise asymmetrically at the R3/R4 cell boundary [34], [35], where they operate to bias a Notch/Delta feedback loop that specifies R3 and R4 photoreceptor cell fates [36]–[38]. In *fz* mutant eyes, the other core proteins never adopt an asymmetric localisation, whereas in *stbm* or *pk^pk-sple^* mutant eyes Fz does become asymmetric, but the onset of asymmetry is delayed [34]. Interestingly, a Fmi∶Fmi-Fz complex can stably localise to junctions in the pupal wing [39]. In the eye, a similar Fmi∶Fmi-Fz complex may ultimately be sufficient to generate asymmetry, when coupled to a Notch-Delta feedback loop to further amplify differences in cell fate. In the absence of Pk/Sple, this complex would form too late to correctly regulate ommatidial rotation and chirality. Perhaps only a weak localisation of Sple to membranes with Stbm is sufficient to bias the orientation of Fz asymmetric localisation, and to do so early enough for correct R3/R4 fate decision and rotation to occur. A similar rationale could also explain the ability of EGFP-SpleΔCaaX to partially rescue the ectopic joints in *pk^pk-sple^* and *pk^sple^* legs, where joints are specified by a Notch/Delta feedback loop, biased by the asymmetric localisation of the core proteins [40], [41].

Interestingly, ommatidial rotation is completely rescued by EGFP-SpleΔCaaX, whilst the rescue of chirality is incomplete. Similarly, EGFP-PkΔCaaX largely rescues the misrotation phenotype. However, only EGFP-Pk, but not EGFP-PkΔCaaX can cause a dominant eye chirality phenotype (indicating a failure to couple to the tissue axes). Thus, ommatidial rotation appears to require less Pk/Sple activity than does coupling to the tissue axis. We propose that when Pk is misexpressed in a wild type background, it displaces Sple from asymmetric complexes, and prevents Sple from mediating coupling to the tissue axes, but that this displacement requires higher levels of Pk activity and is thus enhanced by farnesylation. Therefore, whilst farnesylation promotes membrane association of both Pk and Sple, this is only essential for those aspects of Pk and Sple function that require the highest levels of activity.

## Materials and Methods

### Fly stocks and genetics

Fly stocks are described in FlyBase. *pk^pk-sple13^*, *stbm^6^*, *dsh^V26^* and *dor^8^* are null alleles, and *dsh^1^* is null for planar polarity. *pk^pk1^* and *pk^sple1^* do not express the Pk and Sple isoforms, respectively. RNAi lines are from VDRC (*MVD^IR-24253^*, *Cul-3^IR-109415^*, *skpA^IR-^* ^46605^), NIG (*FNTB^IR-17565R-2^*, *FNTA^IR-2976R-4^*, *Ubc12^IR-7375R-3^*) or DRSC (*skpA^shRNA-HMS00657^*).

Pk and Sple isoforms were tagged at the N-terminus with EGFP, and for the ΔCaaX versions, the last 4 amino acids were deleted. *Myc-pk-PP-HA* and *Myc-pk-PP-HAΔCaaX* were made by inserting 6 myc epitopes at the N-terminus, and deleting the last 4 amino acids as required. Overlap PCR was used to insert a Prescission protease cleavage site (LEVLFQGP) followed by a HA tag (YPYDVPDYA) after amino acid 700 of the Pk open reading frame, which is in an unstructured, poorly conserved region.

*EGFP-pk* and *EGFP-pkΔCaaX* were cloned in *pActP-FRT-polyA-FRT*. *EGFP-sple*, *EGFP-spleΔCaaX*, *Myc-pk-PP-HA* and *Myc-pk-PP-HAΔCaaX* were cloned in a modified *pActP-FRT-polyA-FRT* vector with an *attB* site downstream of the polylinker, and inserted into the *attP2* landing site by øC31 integration. Transgenics were generated by Bestgene and Genetivision.

Mitotic clones were induced using the FLP/FRT system and *Ubx-FLP*. Expression from *pActP* transgenes used *Ubx-FLP* in the wing, or *ey-FLP* in the eye, and for legs the *FRT-polyA-FRT* cassette was flipped out in the germline using *hs-FLP*. For adult wings, *MVD^IR-24253^* was expressed using *MS1096-GAL4* at 18°C and *FNTB^IR-17565R-2^* using *459.2-GAL4* at 29°C. For pupal wings, RNAi lines were expressed using *ptc-GAL4*, with or without *UAS-Dcr2*, and larvae were raised at 18°C and shifted to 25°C at 0 hr APF (*Ubc12* and *skpA* lines) or raised at 25°C and shifted to 29°C at 0 hr APF (*FNTA* and *FNTB* lines). For pupal wing Westerns, RNAi lines were expressed with *MS1096-GAL4*, larvae were raised at 18°C and male prepupae shifted to 29°C for 26 hr at 0 hr AP (*Ubc12*/*Cul-3* blot), or female larvae shifted to 25°C for 28 hr (*skpA* blot).

### Histology and immunolabelling

Adult wings were mounted in GMM and eye sections were prepared as described [42]. Pupal wings were dissected at 28 hr APF at 25°C and imaged as previously [43]. Primary antibodies for immunostaining were rat anti-Pk (recognises both Pk and Sple isoforms, [20]), mouse monoclonal anti-Fmi (DSHB, [44]), rabbit anti-Stbm [45], rat anti-Ecadherin (Ecad, DSHB, [46]), mouse monoclonal anti-Armadillo (Arm, DSHB), rabbit anti-GFP (Abcam), mouse monoclonal anti-Myc 9E10 (DSHB), rabbit anti ß-gal (Cappel) and mouse monoclonal ß-gal (Promega). Phalloidin-A568 was from Molecular Probes.

### Biochemistry and Western analysis

For pupal wing Westerns, 28 hr APF pupal wings were dissected into sample buffer, and 1 pupal wing equivalent was loaded per lane. For MG132 experiments, wing discs from 0 hr APF prepupae were dissected in Schneider's medium containing 10% FCS, and then incubated for 5 hr in Schneider's medium containing 10 µM MG132 in DMSO (or DMSO control). Wings were then transferred into sample buffer.

For phase extractions, total cell lysates from 120 28 hr pupal wings were made in Tris-buffered saline (TBS, 50 mM Tris-HCl, pH 7.5, 150 mM NaCl) containing 1% Triton X-114 (precondensed in TBS) and protease inhibitors (Roche). Lysates were digested for 1 hr at 4°C with 0.5 u Prescission protease (Xerxes), in the presence of 1 mM DTT and 0.5 mM EDTA. Samples were then heated to 37°C for 2 min, and spun at 14K for 2 min at RT. The upper aqueous phase and lower detergent phases were separated and readjusted to TBS/1% Triton X-114, before precipitating with chloroform/methanol and resuspending in sample buffer. Recovery of the protein pellets was confirmed using control antibodies for the aqueous and detergent fractions on Westerns.

For tissue culture, *Myc-pk-PP-HA*, *stbm-EYFP*, *EGFP-pk*, *EGFP-pkΔCaaX* and *Myc-stbm* were cloned in *pMK33ß*. Phase extractions were performed as above. For immunoprecipitations, lysates were made in IP buffer (50 mM Tris-HCl pH 7.5, 150 mM NaCl, 1% Triton X-100, 1× protease inhibitor cocktail (Roche)), and used rabbit anti-GFP serum (Abcam) and protein G sepharose (Xerxes).

Westerns were probed with rat anti-Pk [20], rabbit anti-Fz [47], rabbit anti-GFP (Abcam), mouse monoclonal anti-Myc 9E10 (DHSB), rabbit anti-HA (Abcam), mouse monoclonal anti-Tubulin DM1A (Sigma) and mouse monoclonal anti-Actin AC-40 (Sigma), and imaged on X-ray film or a UVIprochemie gel documentation system (UVItec) for quantitation. Bands from Westerns of at least three biological replicates were quantitated in ImageJ.

## Supporting Information

Figure S1 EGFP-Pk localisation and levels are regulated by Stbm. (A) *stbm^6^* clone, marked by loss of ß-gal staining (red), in pupal wings expressing *ActP-EGFP-pk*, stained for GFP (green). Scale bar 20 µm. (B) Western blot probed with anti-GFP antibody showing EGFP-Pk levels in *ActP-EGFP-pk/+* and *stbm^6^; ActP-EGFP-pk/+* pupal wings, with Actin as loading control. (C) *dor^8^* clone, marked by intracellular accumulation of Fmi (red), stained for Pk (green). No accumulation of Pk is seen inside the clone.
(TIF)

Figure S2 Analysis of Pk prenylation. (A) Schematic of the biosynthetic pathway that produces farnesyl and geranylgeranyl lipid adducts from HMG CoA. Protein names are in grey and the fly genes are in red. The farnesyl-diphosphate farnesyl transferase and geranylgeranyl transferase enzymes consist of alpha and beta subunits, and farnesyl-diphosphate farnesyl transferase and type I geranylgeranyl transferase share their alpha subunits (FNTA). Both these enzymes target CaaX motifs, whilst type II geranylgeranyl transferase targets CC or CaC motifs. Sterol synthesis occurs downstream of farnesyl-PP, but there is no sterol branch in flies. Modified from Santos and Lehmann [48]. (B) Diagram of the Myc-Pk-PP-HA protein, showing the position of the PET/LIM domains of Pk and the inserted Prescission protease (PP) cleavage site and HA tag. (C) *pk^pk-sple13^* clone, marked by loss of ß-gal staining (blue), in wings expressing *ActP-Myc-pk-PP-HA*, stained for Myc (green) and Pk (red). Note asymmetric localisation of Myc-Pk-PP-HA in wild type and *pk^pk-sple^* mutant tissue. Scale bar 20 µm. (D) Adult wing from *pk^pk-sple13^; ActP-Myc-pk-PP-HA/+* fly. (E) Western blot showing lysates from flies expressing *ActP-Myc-pk-PP-HA*, before and after PP cleavage, probed with anti-Myc or anti-HA antibodies. Cleavage produces a large N-terminal fragment tagged with Myc and a small C-terminal fragment tagged with HA (arrows). A non-specific band on the HA blot is marked with an arrowhead.
(TIF)

Figure S3 Partial requirement for Sple farnesylation in the leg. (A–G) Adult legs from wild type (A), *pk^pk-sple13^* (B), *pk^sple1^* (C), *pk^pk-sple13^*; *ActP*-*EGFP-sple/+* (D), *pk^pk-sple13^*; *ActP*-*EGFP-spleΔCaaX/+* (E), *pk^sple1^*; *ActP*-*EGFP-sple/+* (F) and *pk^sple1^*; *ActP*-*EGFP-spleΔCaaX/+* (G) flies. Tarsal segments 1–5 are marked in panel A. Black arrowheads show joints, and grey arrowheads are partial ectopic joints. 50% of *pk^pk-sple13^*; *ActP*-*EGFP-spleΔCaaX/+* and *pk^sple1^*; *ActP*-*EGFP-spleΔCaaX/+* legs contain a partial ectopic joint in T4.
(TIF)

Figure S4 Effects of deleting the prenylation motif of Pk or Sple in the pupal wing. (A,B) 28 hr pupal wings expressing clones of *ActP-EGFP-sple* (A) and *ActP-EGFP-spleΔCaaX* (B), stained for GFP (green) and Fmi (red). EGFP-Sple localises to distal cell edges, in a region of the wing where trichome polarity is reversed. Scale bar 20 µm. (C) *pk^pk-sple13^ stbm^6^* double mutant clone, marked by loss of ß-gal staining (blue), in a 28 hr pupal wing expressing *ActP-EGFP-pkΔCaaX*. Staining is for GFP (green) and Ecad (red). (D) Western blot showing GFP levels relative to Actin levels, in 28 hr pupal wing extracts from *ActP-EGFP-pk/+*,and *ActP-EGFP-pkΔCaaX/+* flies.
(TIF)

Figure S5 *In vitro* analysis of Pk prenylation and binding to Stbm. (A) Phase separation of the HA-tagged C-terminus of Myc-Pk-PP-HA, after cleavage with PP. Cells were transfected with *pAc5.1-Myc-Pk-PP-HA*, with (right) or without (left) *pMK33ß-Stbm-EYFP*. Blots show HA staining of total lysate, aqueous fraction (Aq) or detergent fraction (Det). No prenylation of Pk is observed regardless of whether Stbm is co-transfected. (B) Western blots showing co-IP of Myc-Stbm with EGFP-Pk and EGFP-PkΔCaaX. Note that Jenny et al [6] also showed in GST pulldowns that Pk lacking the last 60 amino acids still binds to Stbm.
(TIF)

Figure S6 SkpA regulates levels of Pk at junctions. (A,B) 28 hr pupal wings expressing *ptc-GAL4/+; skpA^shRNA-HMS00657^*/+, stained for Pk (green), Stbm (red) and either Fmi (blue in A) or Arm (blue in B). Yellow bar marks the *ptc-GAL4* domain. Scale bar 20 µm.
(TIF)

Table S1 RNAi screen of HMG CoA pathway components. In the initial screen, RNAi lines were crossed to *MS1096-GAL4* at 29°C. Those lines where wings could not be mounted due to lethality or where the wings were disrupted (shrivelled or curly) were then crossed to *MS1096-GAL4* at 25°C or *459.2-GAL4* at 29°C. Multiple wing hair phenotypes are common, and are most likely caused by large cells/cell division defects.
(DOC)
