# Supplementary figures and images for: Strabismus Promotes Recruitment and Degradation of Farnesylated Prickle in Drosophila melanogaster Planar Polarity Specification
Source: PLoS Genet. 2013 Jul 18;9(7):e1003654. doi: 10.1371/journal.pgen.1003654 (PMC3715439; doi:10.1371/journal.pgen.1003654)

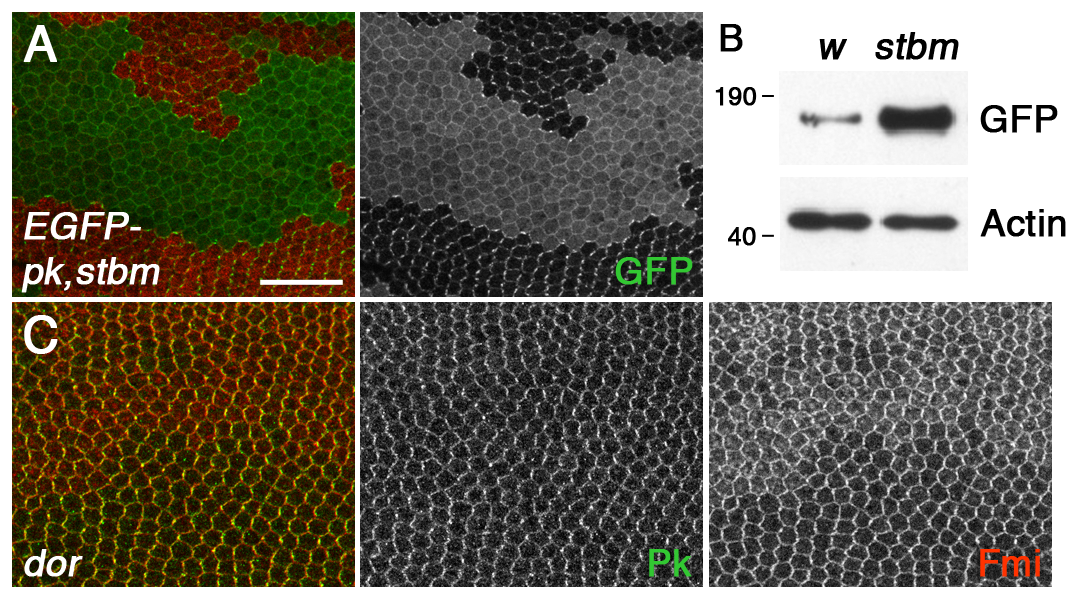

Supplement: Figure S1 — EGFP-Pk localisation and levels are regulated by Stbm. (A) stbm6 clone, marked by loss of ß-gal staining (red), in pupal wings expressing ActP-EGFP-pk, stained for GFP (green). Scale bar 20 µm. (B) Western blot probed with anti-GFP antibody showing EGFP-Pk levels in ActP-EGFP-pk/+ and stbm6; ActP-EGFP-pk/+ pupal wings, with Actin as loading control. (C) dor8 clone, marked by intracellular accumulation of Fmi (red), stained for Pk (green). No accumulation of Pk is seen inside the clone. (TIF) [file pgen.1003654.s001.tif]

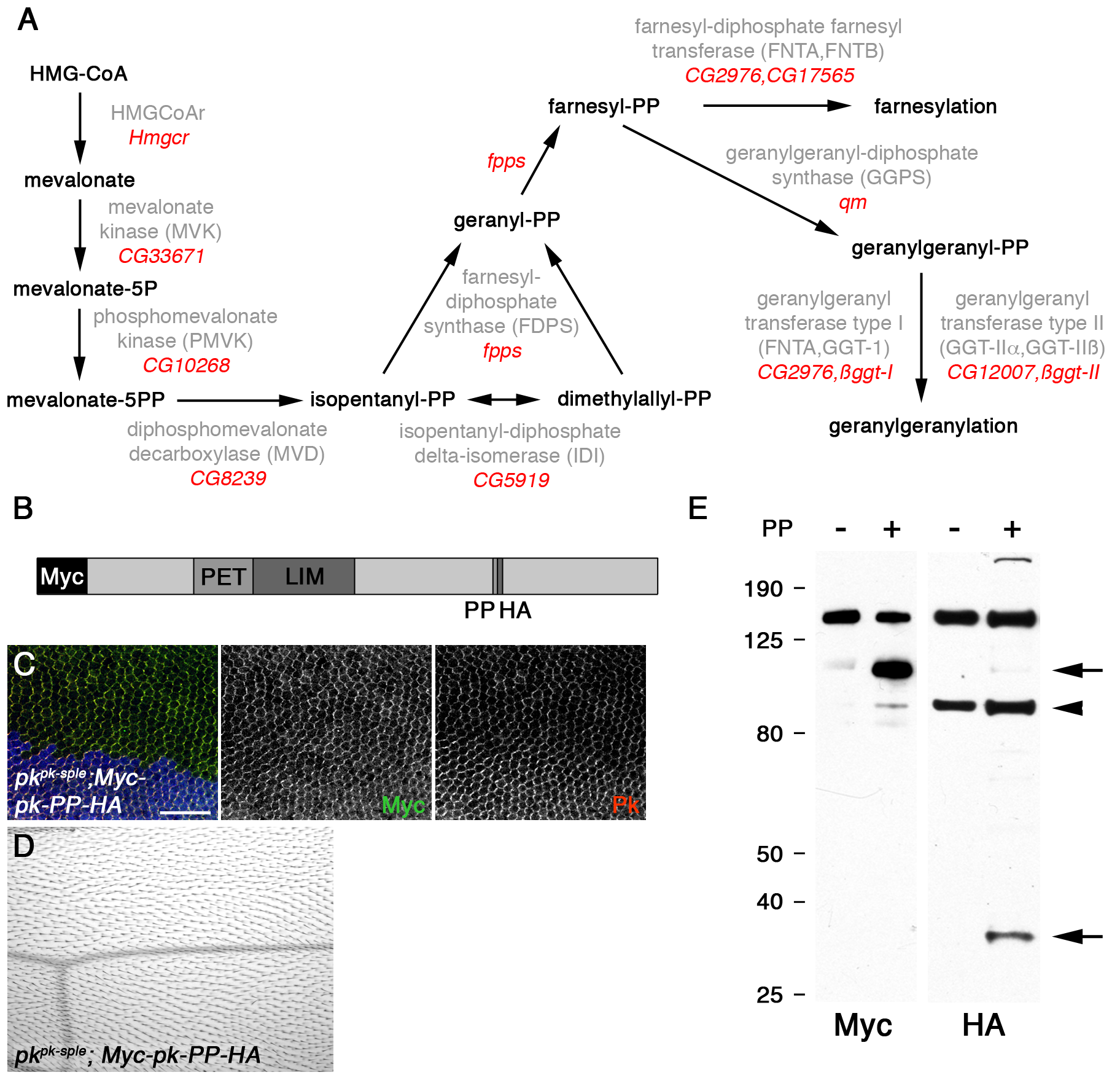

Supplement: Figure S2 — Analysis of Pk prenylation. (A) Schematic of the biosynthetic pathway that produces farnesyl and geranylgeranyl lipid adducts from HMG CoA. Protein names are in grey and the fly genes are in red. The farnesyl-diphosphate farnesyl transferase and geranylgeranyl transferase enzymes consist of alpha and beta subunits, and farnesyl-diphosphate farnesyl transferase and type I geranylgeranyl transferase share their alpha subunits (FNTA). Both these enzymes target CaaX motifs, whilst type II geranylgeranyl transferase targets CC or CaC motifs. Sterol synthesis occurs downstream of farnesyl-PP, but there is no sterol branch in flies. Modified from Santos and Lehmann [48]. (B) Diagram of the Myc-Pk-PP-HA protein, showing the position of the PET/LIM domains of Pk and the inserted Prescission protease (PP) cleavage site and HA tag. (C) pkpk-sple13 clone, marked by loss of ß-gal staining (blue), in wings expressing ActP-Myc-pk-PP-HA, stained for Myc (green) and Pk (red). Note asymmetric localisation of Myc-Pk-PP-HA in wild type and pkpk-sple mutant tissue. Scale bar 20 µm. (D) Adult wing from pkpk-sple13; ActP-Myc-pk-PP-HA/+ fly. (E) Western blot showing lysates from flies expressing ActP-Myc-pk-PP-HA, before and after PP cleavage, probed with anti-Myc or anti-HA antibodies. Cleavage produces a large N-terminal fragment tagged with Myc and a small C-terminal fragment tagged with HA (arrows). A non-specific band on the HA blot is marked with an arrowhead. (TIF) [file pgen.1003654.s002.tif]

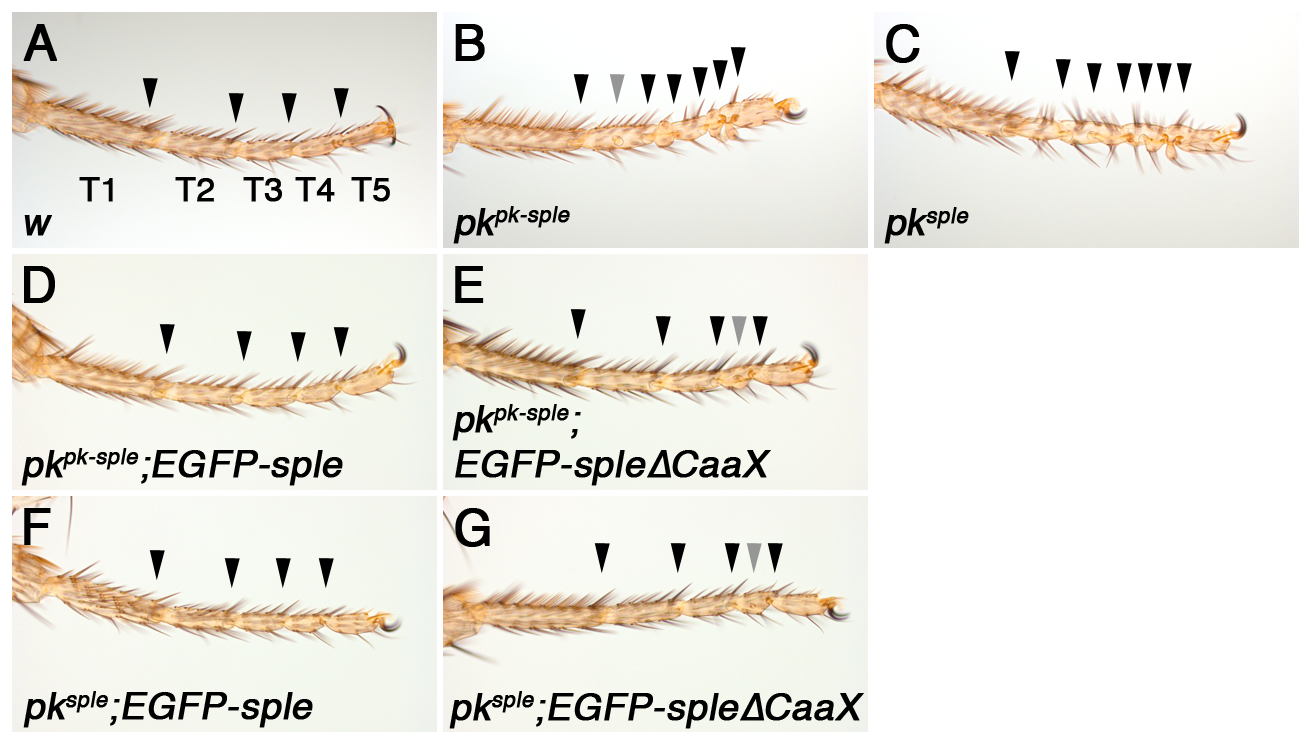

Supplement: Figure S3 — Partial requirement for Sple farnesylation in the leg. (A–G) Adult legs from wild type (A), pkpk-sple13 (B), pksple1 (C), pkpk-sple13; ActP-EGFP-sple/+ (D), pkpk-sple13; ActP-EGFP-spleΔCaaX/+ (E), pksple1; ActP-EGFP-sple/+ (F) and pksple1; ActP-EGFP-spleΔCaaX/+ (G) flies. Tarsal segments 1–5 are marked in panel A. Black arrowheads show joints, and grey arrowheads are partial ectopic joints. 50% of pkpk-sple13; ActP-EGFP-spleΔCaaX/+ and pksple1; ActP-EGFP-spleΔCaaX/+ legs contain a partial ectopic joint in T4. (TIF) [file pgen.1003654.s003.tif]

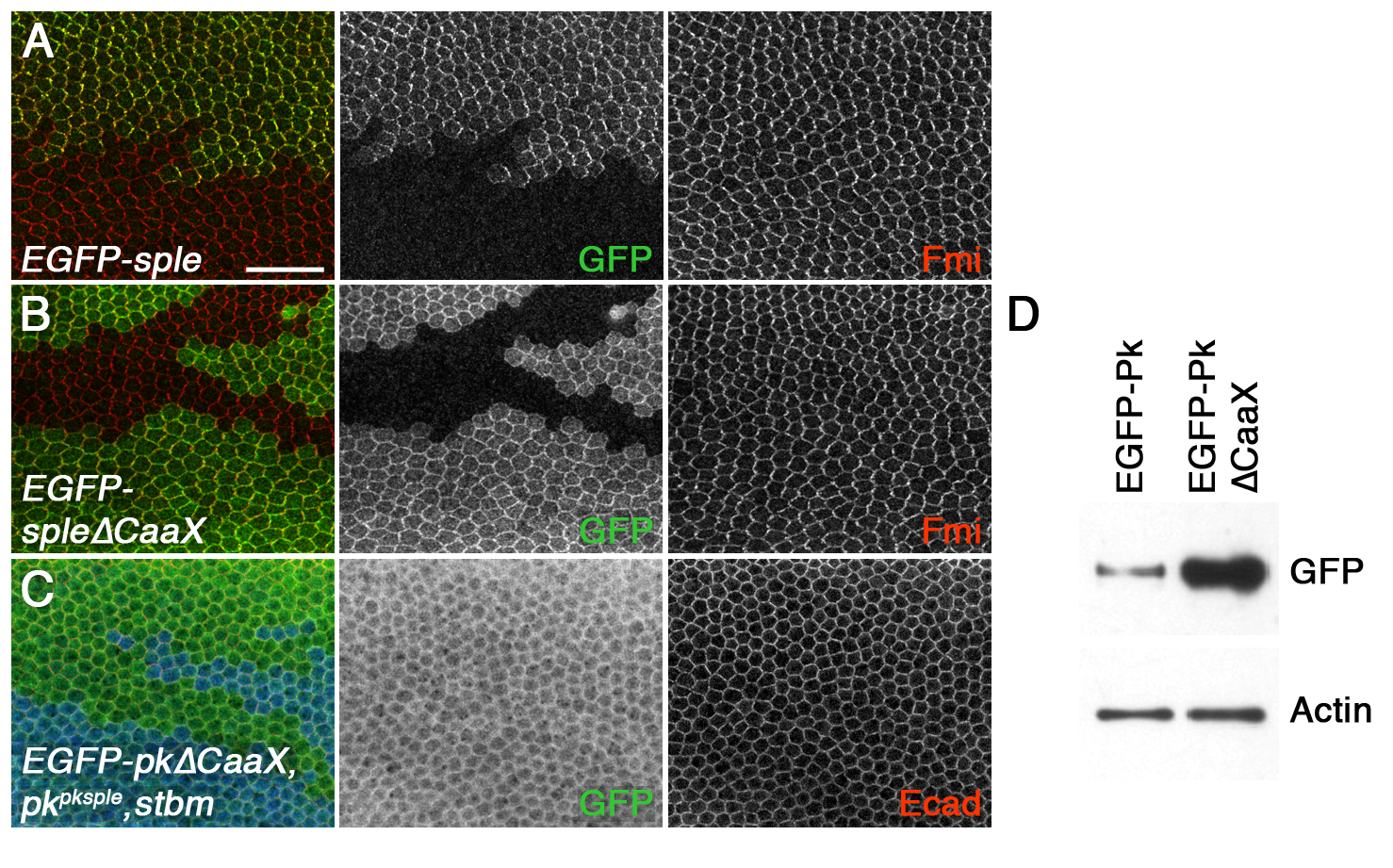

Supplement: Figure S4 — Effects of deleting the prenylation motif of Pk or Sple in the pupal wing. (A,B) 28 hr pupal wings expressing clones of ActP-EGFP-sple (A) and ActP-EGFP-spleΔCaaX (B), stained for GFP (green) and Fmi (red). EGFP-Sple localises to distal cell edges, in a region of the wing where trichome polarity is reversed. Scale bar 20 µm. (C) pkpk-sple13 stbm6 double mutant clone, marked by loss of ß-gal staining (blue), in a 28 hr pupal wing expressing ActP-EGFP-pkΔCaaX. Staining is for GFP (green) and Ecad (red). (D) Western blot showing GFP levels relative to Actin levels, in 28 hr pupal wing extracts from ActP-EGFP-pk/+,and ActP-EGFP-pkΔCaaX/+ flies. (TIF) [file pgen.1003654.s004.tif]

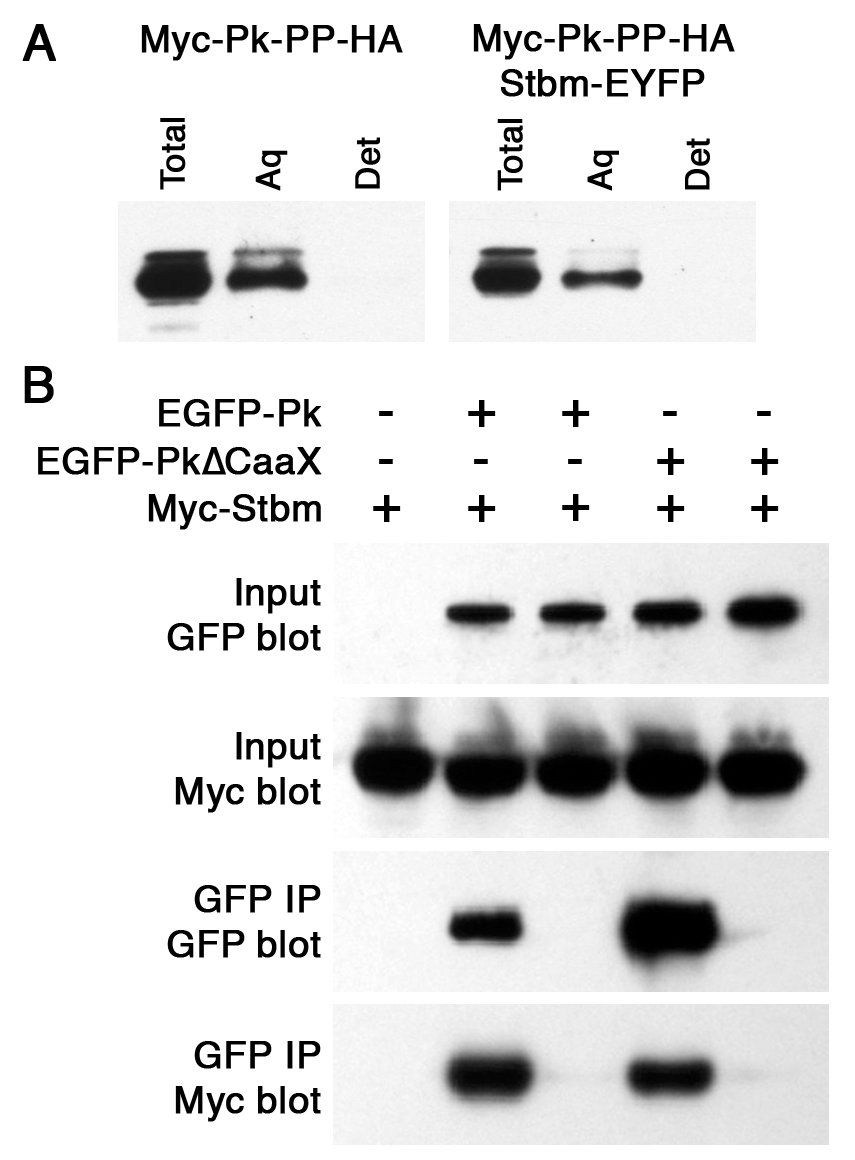

Supplement: Figure S5 — In vitro analysis of Pk prenylation and binding to Stbm. (A) Phase separation of the HA-tagged C-terminus of Myc-Pk-PP-HA, after cleavage with PP. Cells were transfected with pAc5.1-Myc-Pk-PP-HA, with (right) or without (left) pMK33ß-Stbm-EYFP. Blots show HA staining of total lysate, aqueous fraction (Aq) or detergent fraction (Det). No prenylation of Pk is observed regardless of whether Stbm is co-transfected. (B) Western blots showing co-IP of Myc-Stbm with EGFP-Pk and EGFP-PkΔCaaX. Note that Jenny et al [6] also showed in GST pulldowns that Pk lacking the last 60 amino acids still binds to Stbm. (TIF) [file pgen.1003654.s005.tif]

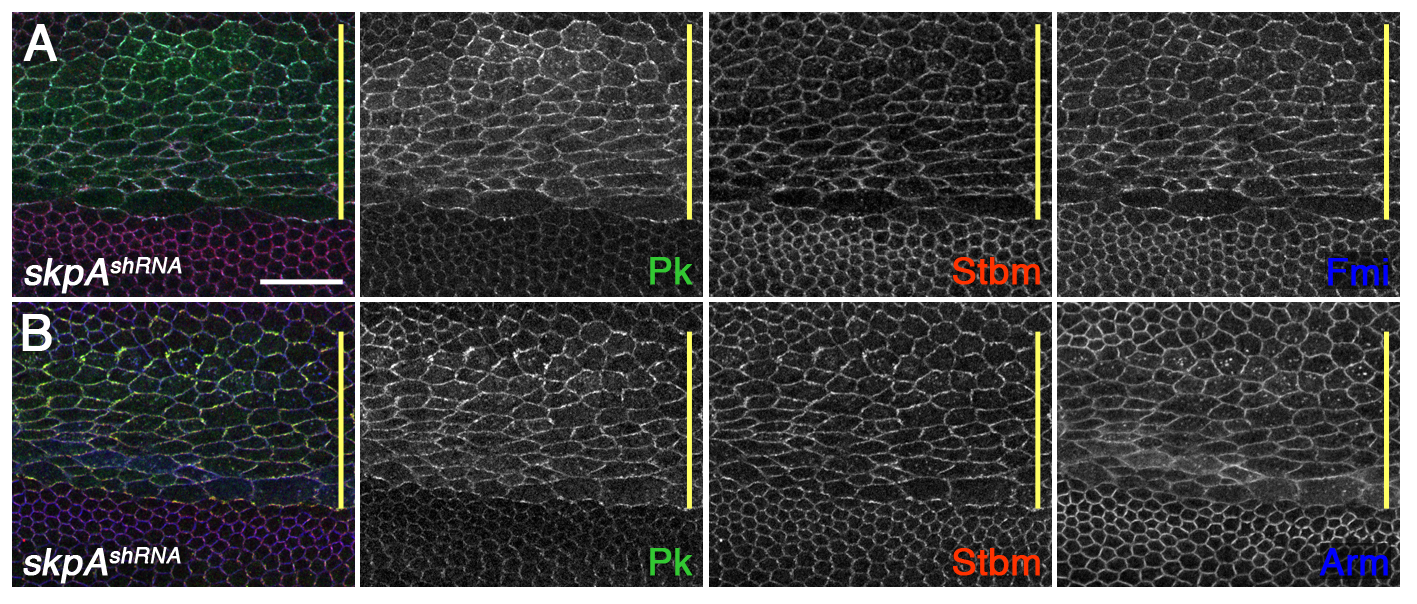

Supplement: Figure S6 — SkpA regulates levels of Pk at junctions. (A,B) 28 hr pupal wings expressing ptc-GAL4/+; skpAshRNA-HMS00657/+, stained for Pk (green), Stbm (red) and either Fmi (blue in A) or Arm (blue in B). Yellow bar marks the ptc-GAL4 domain. Scale bar 20 µm. (TIF) [file pgen.1003654.s006.tif]
