# Supplementary material for: Strabismus Promotes Recruitment and Degradation of Farnesylated Prickle in Drosophila melanogaster Planar Polarity Specification
Source: PLoS Genet. 2013 Jul 18;9(7):e1003654. doi: 10.1371/journal.pgen.1003654 (PMC3715439; doi:10.1371/journal.pgen.1003654)
Supplement: Table S1 — RNAi screen of HMG CoA pathway components. In the initial screen, RNAi lines were crossed to MS1096-GAL4 at 29°C. Those lines where wings could not be mounted due to lethality or where the wings were disrupted (shrivelled or curly) were then crossed to MS1096-GAL4 at 25°C or 459.2-GAL4 at 29°C. Multiple wing hair phenotypes are common, and are most likely caused by large cells/cell division defects. (DOC) [file pgen.1003654.s007.doc]

**Table S1. RNAi screen of HMG CoA pathway components**

| **Gene** | **RNAi line** | **GAL4 driver** | **Phenotype** |
| --- | --- | --- | --- |
| *Hmgcr* | NIG 10367R-1  NIG 10367R-3  VDRC 108617 | *MS1096-GAL4* 29°C  *MS1096-GAL4* 25°C  *459.2-GAL4* 29°C  *MS1096-GAL4* 29°C  *MS1096-GAL4* 25°C  *459.2-GAL4* 29°C  *MS1096-GAL4* 29°C | Wings disrupted  Wings disrupted  Lethal  Wings disrupted, poorly viable  Wings disrupted  Lethal  Lethal |
| *CG33671* | - |  |  |
| *CG10268* | - |  |  |
| *CG8239* | VDRC 24253  VDRC 24254 | *MS1096-GAL4* 29°C  *MS1096-GAL4* 25°C  *459.2-GAL4* 29°C  *MS1096-GAL4* 29°C  *MS1096-GAL4* 25°C  *459.2-GAL4* 29°C | Weak multiple wing hair phenotype  Trichome swirls below vein 5, weak multiple wing hair phenotype  Trichome swirls around vein 4, weak multiple wing hair phenotype  Weak multiple wing hair phenotype  Trichome swirls, weak multiple wing hair phenotype  No phenotype |
| *CG5919* | VDRC 103636 | *MS1096-GAL4* 29°C | Weak multiple wing hair phenotype |
| *fpps* | VDRC 104362 | *MS1096-GAL4* 29°C | Weak multiple wing hair phenotype |
| *CG2976* | NIG 2976R-2  NIG 2976R-4 | *MS1096-GAL4* 29°C  *MS1096-GAL4* 25°C  *459.2-GAL4* 29°C  *MS1096-GAL4* 29°C  *MS1096-GAL4* 25°C  *459.2-GAL4* 29°C | Lethal  Wings disrupted  Lethal  Wings disrupted  Wings disrupted  Lethal |
| *CG17565* | VDRC 32951  VDRC 32952  NIG 17565R-2  NIG 17565R-5 | *MS1096-GAL4* 29°C  *MS1096-GAL4* 29°C  *MS1096-GAL4* 29°C  *MS1096-GAL4* 25°C  *459.2-GAL4* 29°C  *MS1096-GAL4* 29°C | No phenotype  No phenotype  Wings disrupted  Trichome swirls below vein 4  Trichome swirls below vein 4  No phenotype |
| *qm* | VDRC 47000  VDRC 47001 | *MS1096-GAL4* 29°C  *MS1096-GAL4* 25°C  *459.2-GAL4* 29°C  *MS1096-GAL4* 29°C  *MS1096-GAL4* 25°C  *459.2-GAL4* 29°C | Weak multiple wing hair phenotype, some loss of margin bristles  No phenotype  Lethal  Strong multiple wing hair phenotype, some loss of margin bristles  No phenotype  Lethal |

| **Gene** | **RNAi line** | **GAL4 driver** | **Phenotype** |
| --- | --- | --- | --- |
| *ßggt-I* | VDRC 24759 | *MS1096-GAL4* 29°C  *MS1096-GAL4* 25°C  *459.2-GAL4* 29°C | Lethal  Wings disrupted  No phenotype |
| *CG12007* | VDRC 20137  VDRC 47403  VDRC 47404  VDRC 47405  VDRC 50390  NIG 12007R-2  NIG 12007R-4 | *MS1096-GAL4* 29°C  *MS1096-GAL4* 29°C  *MS1096-GAL4* 25°C  *459.2-GAL4* 29°C  *MS1096-GAL4* 29°C  *459.2-GAL4* 29°C  *MS1096-GAL4* 29°C  *459.2-GAL4* 29°C  *MS1096-GAL4* 29°C  *459.2-GAL4* 29°C  *MS1096-GAL4* 29°C  *MS1096-GAL4* 29°C  *MS1096-GAL4* 25°C  *459.2-GAL4* 29°C | No phenotype  Lethal  No phenotype  Lethal  No phenotype  Lethal  No phenotype  Lethal  No phenotype  Lethal  No phenotype  Wings disrupted  No phenotype  Lethal |
| *ßggt-II* | VDRC 33386  NIG 18627R-2  NIG 18627R-3 | *MS1096-GAL4* 29°C  *MS1096-GAL4* 25°C  *459.2-GAL4* 29°C  *MS1096-GAL4* 29°C  *MS1096-GAL4* 25°C  *459.2-GAL4* 29°C  *MS1096-GAL4* 29°C  *MS1096-GAL4* 25°C  *459.2-GAL4* 29°C | Wings disrupted  Wings disrupted  Lethal  Lethal  Wings disrupted  Lethal  Lethal  Wings disrupted  Lethal |

In the initial screen, RNAi lines were crossed to *MS1096-GAL4* at 29°C. Those lines where wings could not be mounted due to lethality or where the wings were disrupted (shrivelled or curly) were then crossed to *MS1096-GAL4* at 25°C or *459.2-GAL4* at 29°C. Multiple wing hair phenotypes are common, and are most likely caused by large cells/cell division defects.
